# Editorial: From social wires to neurobiological connections: a neuropsychobiological focus on parent-child interaction

**DOI:** 10.3389/fnbeh.2024.1487555

**Published:** 2024-09-20

**Authors:** Livio Provenzi, Marina Fuertes, Isabella L. C. Mariani Wigley, Sarah Nazzari

**Affiliations:** ^1^Department of Brain and Behavioral Neuroscience, University of Pavia, Pavia, Italy; ^2^Developmental Psychobiology Lab, IRCCS Mondino Foundation, Pavia, Italy; ^3^Centro de Psicologia, University of Porto, Porto, Portugal; ^4^Escola Superior de Educação de Lisboa, Lisbon, Portugal; ^5^Department of Developmental and Social Psychology, University of Padua, Padua, Italy

**Keywords:** parenting, infant, interaction, emotion development, stress regulation, neuroscience, psychobiology, thermal imaging

## Connections between humans shaping developmental connections within humans

The early social interactions between parents and children are foundational to both the neurobiological development of the child and the establishment of parental mental health and wellbeing. These connections, forged through daily interactions, are much more than mere exchanges of affection or communication; they are integral to shaping the brain and behavior of both the parent and the child. This intricate interplay serves as a driving force behind critical developmental processes (Provenzi et al., 2018; Tronick and Hunter, 2024). Through day-to-day interactions sensitive and responsive parents shape several key aspects of child development. These exchanges have a direct impact on infants' brain development, emotional regulation, social information processing, and attachment formation (Labella et al., 2024). For example, when parents respond sensitively to their child's needs, they not only support the child emotional regulation; rather they also reinforce neural pathways associated with secure attachment and adaptive stress responses (Madigan et al., 2024). Conversely, inconsistent or negative interactions can disrupt these processes, leading to challenges in emotional regulation and social functioning (Cooke et al., 2022; Nazzari et al., 2022). The biobehavioral processes that emerge from these interactions—including behavioral responses, emotional regulation, and cognitive representations—are essential for shaping the developmental trajectory of the child and the psychological wellbeing of the parent. These processes are interconnected in ways that reflect the deeply intertwined nature of social and neurobiological connections within the parent-child relationship (Tronick et al., 2020). Understanding these early interactions is crucial for a comprehensive grasp of both child development and parental mental health.

As research in pediatric psychology advances, it becomes increasingly evident that the social bonds between parents and children are central to forming the neurobiological framework that supports emotional and cognitive processes in both individuals. This understanding emphasizes the importance of investigating how early relational experiences shape neurobiological outcomes and, conversely, how these outcomes influence ongoing relational dynamics. In light of these insights, it is imperative to develop a research framework within pediatric psychology that recognizes the significance of early parent-child connections. Such a framework should not only focus on the immediate effects of these interactions but also consider their long-term implications for both child development and parental mental health. By valuing these early connections as a critical area of study, researchers can gather scientific evidence that informs care practices and supports the development of interventions aimed at enhancing both parental and child wellbeing. This comprehensive approach will ultimately contribute to a more nuanced understanding of how early social interactions shape the neurobiological and psychological landscapes of both parents and children.

In line with this approach, we have curated the Research Topic entitled “*From social wires to neurobiological connections: a neuropsychobiological focus on parent-child interaction*” to initiate a paradigm shift in pediatric psychology. This Research Topic provides a promising glimpse into how integrating social and neurobiological perspectives can deepen our understanding of child development and parental mental health. The contributions gathered here are partial and preliminary, yet they highlight the emerging insights and methodologies that underscore the significance of these early connections. We believe that through collaboration among researchers, clinicians, and families, we can advance our knowledge and enhance our ability to support children effectively. This interdisciplinary approach will pave the way for improved evidence-based practices and targeted interventions that address both psychological and neurobiological needs.

## What can you find in this Research Topic?

### How parenting contributes to shaping emotion development

Gonçalves et al. examine how attachment security influences infants' attention to emotional expressions, providing compelling evidence that these early social connections significantly affect how infants process and respond to social stimuli. Their findings reveal that securely attached infants are better equipped to handle new emotional information, while insecurely attached infants display signs of either hypervigilance or avoidance. These differences underscore the profound influence of social connections with caregivers on the neurobiological mechanisms underlying emotion regulation. Fuertes and Gonçalves explore the themes of continuity and discontinuity in mother-infant interactions, particularly in the context of prematurity. Their research highlights that the stability of these social connections—or the lack thereof—can have significant implications for the neurobiological and psychological development of both the child and the parent. In preterm dyads, the interactions tend to be more stable, but they may lack the necessary adaptability to respond effectively to developmental challenges, emphasizing the importance of flexible and responsive parenting in fostering healthy neurobiological connections.

### How caregiving deprivation can detrimentally shape brain development

Oliveira reviews the impact of early caregiving adversity on brain development, drawing a direct correlation between the quality of early social connections and subsequent neurobiological outcomes. The review synthesizes evidence demonstrating that children exposed to adverse caregiving environments, such as institutionalization, experience significant alterations in brain structure and function, which impair their ability to form healthy attachments. This work highlights the critical role of nurturing social connections in safeguarding and promoting healthy neurobiological development, especially in vulnerable populations.

### How dyadic thermal imaging can help us understand early social connections

Nazzari et al. introduce infrared thermal imaging (ITI) as a novel, non-invasive method for studying parent-infant co-regulation, with a focus on the physiological aspects of these social connections. Their research shows that ITI can detect subtle physiological changes during parent-child interactions, offering new insights into how these social bonds shape the functioning of the autonomic nervous system in both parents and infants. The ability to observe these connections in real-time and naturalistic settings presents exciting new opportunities for understanding the physiological foundations of the parent-child bond.

### How mindful parenting can be neuroprotective for child development

Passaquindici et al. investigate the impact of mindfulness on mother-infant attunement, providing evidence that fostering the social connection between parent and child can reinforce neurobiological connections. Their research suggests that mindfulness practices improve emotional and behavioral synchronization between mother and infant and contribute to better mental health outcomes for the parent. This dual benefit underscores the deeply interconnected nature of social and neurobiological connections in parent-child interactions, highlighting the potential of mindfulness as a tool for enhancing these vital bonds.

## Conclusions

The studies included in this Research Topic are concordant in underscoring the significant role of social connections between parents and children in shaping neurobiological development. This body of research highlights that parent-child interactions are not merely transient or isolated moments of connection; rather, they form the foundational building blocks of a complex neurobiological framework that influences emotional and cognitive functioning throughout an individual's life. These early interactions between parents and children are critical for the development of neural circuits that govern emotional regulation, social behavior, and cognitive processes (Belsky and De Haan, 2011; Mariani Wigley et al., 2022). By focusing on the intricacies of parent-child interactions, researchers are able to uncover valuable insights into how these early experiences shape not only child development but also parental mental health. Understanding the ways in which social bonds influence neurobiological processes allows for a more nuanced view of how these interactions impact long-term emotional and cognitive outcomes. As research in this area grows, it will be increasingly needed to embrace a comprehensive view of parent-child interactions, integrating social and neurobiological perspectives. Such integrated approach is essential for developing interventions that not only address immediate needs but also contribute to long-term emotional and cognitive health. By bridging these fields, we can better support families and improve developmental outcomes for children, ultimately enhancing the overall quality of life for both parents and their children.
